# Anti-influenza Activity of a Novel Polyoxometalate Derivative (POM-4960)

**Published:** 2012

**Authors:** Seyed Masoud Hosseini, Elham Amini, Masoumeh Tavassoti Kheiri, Parvaneh Mehrbod, Mahsa Shahidi, Ebrahim Zabihi

**Affiliations:** 1 *Department of Microbiology, Faculty of Biological Sciences, Shahid Beheshti University, Tehran, Iran *; 2 *Influenza Unit, Pasteur Institute of IRAN, Tehran, Iran *; 3 *Cellular and Molecular Biology Research Center, Babol University of Medical Sciences, Babol, Iran *

**Keywords:** POM, influenza virus, RT-PCR, immunofluorescent, MTT

## Abstract

There are many effective chemotherapeutic agents used in influenza disease which some of them inhibit virus replication by interfering with FluV (influenza virus) viral binding or its penetration into cell membrane. A series of polyoxometalates compounds such as POM-523 and PM-504 have been synthesized and have showed inhibitory effects on viruses. In this study we examined anti influenza activity of a novel polyoxometalate derivative (POM-4960) synthesized in the Faculty of Chemistry of Damghan University of Basic Sciences. To evaluate the anti-influenza activity of POM, following the treatment of FluV with POM at different temperatures and incubation periods, viral titer reduction was assessed by haemaglutination assay (HA). The 3-(4,5- dimethylthiazol-2-yl)-2,5-diphenyltetrazolium bromide (MTT) assay was used to determine TCID50 (tissue culture infective dose) of virus, CC50 (median cytotoxic concentration) of POM, protection percentage and antiviral activity of POM in cell culture. RT-PCR and direct Immunofluorescent assays were performed to evaluate the effect of POM on viral infection and viral RNA load, respectively.

POM reduced HA titer near to zero in all cell culture specimens and showed high protection against viral infection of the cells. Reduction in viral infection was confirmed by RT-PCR and Immunofluorescent staining methods. Moreover, this POM derivative has a dual (cumulative) effect on attachment and penetration inhibition compared to other POM’s with just one inhibitory effect. POM-4960 could be considered as a powerful anti-influenza agent with low toxicity and high antiviral potency.

Influenza virus type A is categorized into different subtypes based on its two important surface glycoproteins. Hemagglutinin (HA) is associated with the virus affinity to a host cell via a receptor, prior to its infection, while neuraminidase (NA) is involved in the viral escape from the host cell after infection. Currently, two subtypes are circulating in humans; H1N1 and H3N2. As far as we have known, antigenic shift and drift of influenza A are closely related to the pandemics and lack of long lasting vaccines. For these reasons, numerous investigations on anti influenza drugs, antiseptics and disinfectants have been conducted and still ongoing (1,2). Besides the uncoating inhibitors and NA inhibitors, there are several compounds that prevent viral attachment and its penetration by attacking the HA as a novel target in FluV replication (3). These inhibitors are specially interesting because they interfere with the first steps of viral infection. 

Influenza virus binds to the host cell membrane using HA protein which consequently is cleaved by host cell protease to HA1 and HA2 molecules. After binding of the virion to the host cell membrane by means of the ‘head’ portion of HA1 molecule, the conformation of HA molecule changes and the cleft edge of HA2 peptide appears and attaches to the cell membrane. The edge of HA2 peptide makes the envelope of virion and the host cell membrane fuse (4). In 1995 it was reported that a series of polyoxometalate compounds could block the fusion of some myxoviruses (5). 

Polyoxometalates (POM) are soluble inorganic cluster-like compounds formed principally of an oxide anion and early transition metal cations (6). By using a variety of virus and POMs in time-of-addition studies, it was determined that the ability of POMs to block the adsorption process may hinge on the structure, shape or charge of the compound. A POM derivative named hs-058 inhibited FluV A and RSV (Respiratory Syncytial Virus) replication when added after virus adsorption, but it did not inhibit FluV A replication when added during virus adsorption (6). The mechanism of anti FluV activity of POM depends on the nature and structure of the POM. For instance, POM-523 was shown to inhibit the membrane fusion of virus, whereas PM-504 inhibited the binding of FluV to Madine-Darby Canine Kidney (MDCK) cells. The cell membrane surface is normally covered by negatively charged polymers. The viral particle is attracted electrostatically to the cell membrane and binds to a specific receptor via the HA molecule (7). Negatively charged polymers, such as sulphated polysaccharides, lignans, polyphenoles and some kind of polyoxometalates are thought to be non-specifically disturbing the approach and binding the virions to their receptors (8,9). 

It has been shown that POM-523 could inhibit the opening of the head of HA1 during the conformational change of the HA molecule. The fluorescence dequenching test has been performed to monitor the fusion of the virus envelope with cell membrane, and the mechanism of POM-504 activity was found out to be binding inhibition of FluV to MDCK cells (10). In this study a new synthesized POM (POM-4960) was tested for its antiviral activity effects on FluV A infection in MDCK cell line. 

## Materials and Methods

**Viruses**

Influenza A /New Caledonia/20/99 (H1N1) was used throughout the experiments. The virus was prepared by several passage of standard virus obtaining from National Institute for Biological Standards and Control (NIBSC,UK). It was propagated in MDCK cells overlaying with DMEM medium without FBS containing 2 µg Trypsin-TPCK (Tosylamide, Phenylethyl Chloromethyl Keton-treated Trypsin), 100 mg/ml streptomycin and 100 IU/ml penicillin G (Sigma-Aldrich, St. Louis, USA)

**Cell culture**

 MDCK cells were used for virus titration and antiviral assays of the tested compounds. Cells were cultured in Dulbecco’s Modified Eagle’s Medium (DMEM) supplemented with 100 mg/ml streptomycin (Sigma-Aldrich, St. Louis, USA), 100 IU/ml penicillin G, and 10% heat-inactivated fetal bovine serum (FBS) (Gibco, Gaithersburg, USA) at 37 °C in a humidified 5% CO_2_ incubator. Cell maintenance medium for making virus pools and performing antiviral assays consisted of DMEM without FBS and containing 2 mg/ml Trypsin-EDTA (Ethylene Diamine Tetra-acetic Acid).

**Test compound**

The newly designed POM-4960 with formula (P2W18O62)^6-^ was especially provided by the Faculty of Chemistry - Damghan University of Basic Sciences (11,12). The microbial and hemolytical properties of the compound were checked and their different concentrations were prepared in culture medium.

**Colorimetric MTT assay**

The MTT assay is based on the evaluation of the cells viability using the optical density of produced formazan by active mitochondria as described previously by Raphael (13,14). Briefly, the media of the cells cultured in a 96-well plate was removed and 100 µl of Tetrazolium salt, MTT (3-(4,5-dimethylthiazol-2-yl)-2,5-diphenyltetrazoli-um bromide, Sigma-Aldrich St Louis, USA), was added to each well and incubated at 37°C for 2 h. Then, the reduced MTT dye (formazan precipitate) was dissolved and extracted from the cells by adding 100 µl isopropanol to each well. The absorbance of the formazan was measured at two different wave lengths (540 and 690 nm). 

**Determination of TCID**_50_

The viral infectivity was determined using a 2-day culture of MDCK cells. Ten-fold serial dilutions of virus in the cell maintenance medium were inoculated into the confluent cell culture, which was further incubated at 37 °C for 24 h. In 2 days after infection a typical cytopathic effect (CPE) of FluV was observed and the TCID_50_ was determined by the method of Reed and Muench and Karber (15,16).

**Determination of CC**_50_ **, MTC and MIC of POM-4960**

The cytotoxicity of compound against MDCK cells was evaluated and reported as 50% cytotoxicity concentration (CC_50_), maximum tolerable concentration (MTC) and minimum effective concentration 99 (MEC_99_). The MDCK cells were exposed to different concentrations of POM-4960 in cell maintenance medium without trypsin at 37°C for 24 h. The viabilities of the cells were assessed by the MTT method. The MTC, MEC_99_, and CC_50 _of POM-4960 were determined using SPSS software.

**Antiviral assay**

For antiviral assay, TCID_50_ concentrations of the virus in cell maintenance medium was exposed to CC_50_ of POM-4960 at different intervals (15, 30, 45 min, 1, 4, 6 and 8 h) with different temperatures (4, 23 and 37°C). Also hemagglutination assay was performed to assess viral titer reduction. In order to elucidate the POM-4960 antiviral mechanism of action against FluV-A, the compound was added to host cells before, during and after viral infection at 3 different procedures.

**Procedure I (exposure before the infection)**

A 100 µl of virus exposed to maximum tolerable concentration of POM-4960 for 1h at 37°C, was added to the confluent monolayer of MDCK cells in 96-well plates. Then the supernatants were removed, the cells were washed with PBS and 100 µl/well TPCK containing cell maintenance medium was added (POM-4960+FluV).

**Procedure II (exposure during the infection)**

Following inoculation of 100 TCID_50_ of virus to the confluent cells (100µl/well) and incubation at 37°C for 1 h the supernatant (containing virus suspension) was discarded and the cells were rinsed with PBS. A 100 µl of maximum tolerable concentration of POM-4960 prepared in DMEM containing TPCK was added to each well.

**Procedure III (exposure before & after the infection)**

A 100 TCID_50_ of virus was treated by maximum tolerable concentration of POM-4960 for 1 h at 37°C. A 100 µl of this mixture was added to each well and incubated in 37°C for another 1 h. Then the cells were washed with PBS and 100 µl of POM-4960 diluted in TPCK containing medium 

 was added to each well. All these 3 experimental procedures were followed by 48 h incubation at 37^°^C. Then, the cell supernatants were removed for complementary tests such as virus titration (HA test) and the monolayer cells were used for MTT viability test. In MTT assay, the viability of infected and non-infected cells was presented as the percentage of protection using the following equation: [(OD*T*)*V *- (OD*C*)*V*]/ [(OD*C*)*M *- (OD*C*)*V*] × 100 

 where (OD*T*)*V *, (OD*C*)*V*, and (OD*C*)*M* indicate the differential absorbance (optical density ^2^) of the test sample, the virus-infected control (no compound), and the mock-infected control (no virus and no compound), respectively (14).

**Hemagglutination assay**

Serial twofold dilutions of supernatant of infected cells (100 µl) were prepared using PBS in a 96-well plate. A 50 µl of 0.5% chicken red blood cells (cRBCs) was added to each well. After 1 h incubation at 4°C, hemagglutination and precipitation of red blood cells indicated the presence or absence of the virus, respectively.

**RNA extraction and RT-PCR**

Supernatant of infected cells (100µl) was used for extraction of viral genomic RNA using AccuPrep^®^ Viral RNA Extraction Kit (Bioneer, Korea). Using this kit, RNA was bound to glass fibers fixed in a column and finally was isolated and eluted in the 50µl elution buffer. Since the influenza A virus has been used in this study, influenza B virus was added to the samples as internal control.

**Semi-Quantitative PCR analysis**

Complementary DNA was synthesized from 10 μl viral RNA using Superscript^TM^III First-Strand Synthesis System for RT-PCR Kit (Invitrogen, CA). This kit uses random hexamer primers and a mixture of dNTP and buffer would be incubated at 56˚C for 5 min. The PCR reaction was performed according to the study of Shahidi et al. (17). Primers used in this study were designed for amplification of NP gene (Nucleoprotein) of influenza B and H_1_ (heamagglutinin) gene of influenza A. Finally PCR products (amplicons) were run in 1.5% agarose gel containing ethidium bromide and were detected on UV transluminator after electrophoresis. The PCR products were measured semi-quantitavely by determining band density ratio using “Image Tool” software.

**Antibody**

Influenza A direct FITC-conjugated antibody was prepared in goat serum and then labeled with FITC (UsBiological, Massachusetts). This antibody was highly specific to influenza A H1N1 and its best dilution for this experiment was determined to be 1:50 in blocking buffer. 

**Direct immunofluorescent assay**

Confluent MDCK cells grown on chamber-slides were infected by tenfold dilution of the virus which was previously treated with EMCC of POM-4960 and incubated for 1 h at 37^°^C with 5% CO_2_. Ten-fold dilution of untreated virus inoculated to MDCK cells was used for negative control. After 1 h the cells were rinsed with PBS and overlaid with cell maintenance medium (DMEM medium containing 2 µg/ml Trypsin-TPCK, 100 mg/ml streptomycin and 100 IU/ml penicillin G) the cells were incubated for another 1 h for virus attachment. Then the chamber compartments were separated from the glass slide and the cells were rinsed with PBS. Following 10 min fixation in cold acetone and washing the cells with PBS, blocking buffer (FBS+PBS, 1:10) was used. Finally staining was performed by adding 200 µl FITC-conjugated antibody and after 1 h incubation in the dark at room temperature, the slides were examined by fluorescent microscope (Nikon E200) equipped with fluorescein filter (18).

## Results

**Determination of CC**_50_ **, MTC and MEC**_99_ **of POM-4960**

**Fig 1:**
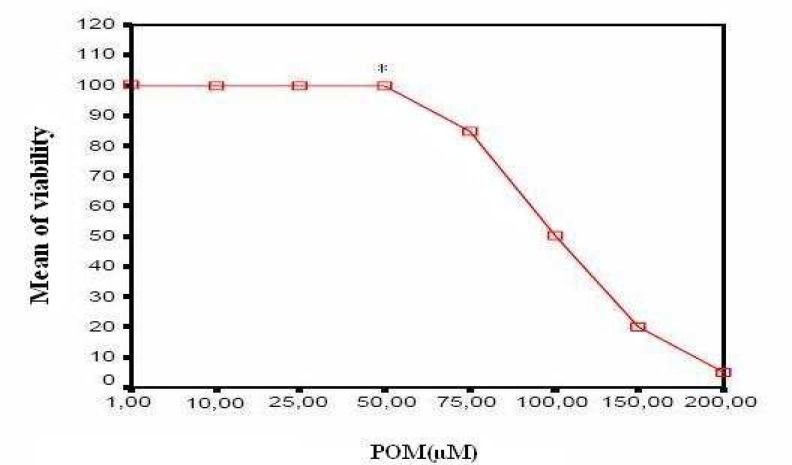
Correlation between dose of POM-4960 and its cytotoxicity in MHCD cell line The median cytotoxic concentration (CC_50_) and maximum tolerable concentration (MTC) of POM-4960 were calculated as 100µM.and 50 µM respectively*: Maximum tolerable concentration

 Following inoculation of the cells with different concentration of POM-4960, the viability of the cells was measured by MTT assay. The results indicate that POM-4960 had no serious cytotoxic effect on MDCK cells at concentration up to 50 µM/ml (Fig.1). The CC_50_ of POM-4960 was calculated to be 100 µM in MDCK cell line. Maximum tolerable concentration of the POM-4960 was determined as 50 µM/ml using MTT standard curve which showed no significant cytopathic effect (CPE) on the cells while obviously reducing the viral infectivity (Table 1)**.** The MEC_99_ (minimum effective concentration with 99% effectiveness) was determined as 10 µM/ml.

**Table 1:** MDCK cells viability after exposure to different concentration of POM-4960 on MDCK cells (MTT assay)

**POM-4960 concentration** **(µM/ml)**	**OD Mean± SD**
1	0.650000**±**0.02160
10	0.649000**±**0.01826
25	0.647500**±**0.01732
50	0.647250**±**0.01893
75	0.550250±0.04787*
100	0.328250±0.02363*
150	0.130250±0.01708*
200	0.032750±0.02217*

Confluent MDCK cells were exposed to different concentrations of POM-4960 for 48 h. The differential absorbance (OD _540-630nm_) of blue dissolved formazane dye was measured in MTT assay. Values are averages of four independent examinations.

٭ Significantly different compared to untreated group* (p<0.0001)*.

**Antiviral assay**

Hemagglutination assay showed that POM-4960 can effectively suppress viral activity after 1 h. Incubation at 37°C. MTT assay determined the viability of mock-infected and normally infected cells in all three experimental procedures following 48 h incubation at 37^°^C (Table 2). Comparative analysis of MTT results in POM-4960 treated and untreated samples using Anova test (Tukey post hoc test) clearly showed significant difference in cell viability (p<0.0001) among the groups. Protection percentage for each group was obtained by analysis of MTT results using SPSS software version 11.0 and presented in (Table 3).

**Table 2:** Viability of MDCK cells infected by FluV exposed to POM-4960 with three differnet procedures (before,during, and after infection

**Sample**	**OD Mean± SD**
POM (50 µM) + virus	0.50900**±**0.018257*
virus → POM (50 µM)	0.498825**±**0.015157*
POM (50 µM)+virus → POM (50 µM)	0.522195**±**0.002858*
Cell control group	0.645250**±**0.120787
Virus control group	0.186125**±**0.002175

MTD concentrations of POM-4960 were made in the cell culture media at the presence of FluV with three different procedures (before, after, and during infection). Viability of the cells were measured as differential OD_540-630nm_ of produced formazan dye (MTT assay). Values are averages of four independent examinations.

FluV; influenza virus, POM-4960+virus; exposure to POM-4960 before FluV infection, Virus → POM - 4960(50µM); exposure to POM-4960 after FluV infection, POM(50µM) + virus **→** POM - 4960(50µM); exposure to POM-4960 after infection with POM-4960 treated FluV.

* Significantly different from values obtained for POM treated compared to untreated sample *(p<0.*0001).

**Table 3:** Protection percentage of POM-4960 against virus infection in each procedure

**Exposure procedure**	**Protection percentage** *(%)
POM (50 µM) + virus	75
virus → POM (50 µM)	73
POM (50 µM)+virus → POM (50 µM)	78

* Protection percentage was calculated using differential OD_540-630nm_ of formazan (MTT test) in protection percentage formula (see the text for details). FluV; influenza virus, POM-4960+virus; exposure to POM-4960 before FluV infection, Virus → POM - 4960(50µM); exposure to POM-4960 after FluV infection, POM(50µM) + virus → POM - 4960(50µM); exposure to POM-4960 after infection with POM-4960 treated FluV.

**Hemagglutination assay**

Supernatant of cells was removed and used for HA test in all three experimental procedures of mechanism investigation. The result indicates that POM-4960 could remarkably inhibit the virus hemagglutination and eventually turn the HA titer into zero (table 4).

**Table 4:** Hemagglutination of virus in supernatants of cells exposed to POM-4960 (with procedures I, II, III) compared to control group (normal FluV MDCK cell infection

**Group**	**HA unit**
Control (10^-1^)	512
Procedure I	0*
Procedure II	0*
Procedure III	0*

* Hemagglutination of virus in all procedures (I, II, III)reduced to 0 HA unit. The experiments Values are averages of four independent examinations for HA assay. FluV; influenza virus, Procedure I: {POM-4960+virus; exposure to POM-4960 before FluV infection}, Procedure II: {Virus POM – 4960(50µm); exposure to POM-4960 after FluV infection}, Procedure III: {POM (50µM)+virus POM -4960(50µM); exposure to POM-4960 after infection with POM-4960 treated FluV}.

**RNA extraction and semi-quantitative RT-PCR**

**Fig 2:**
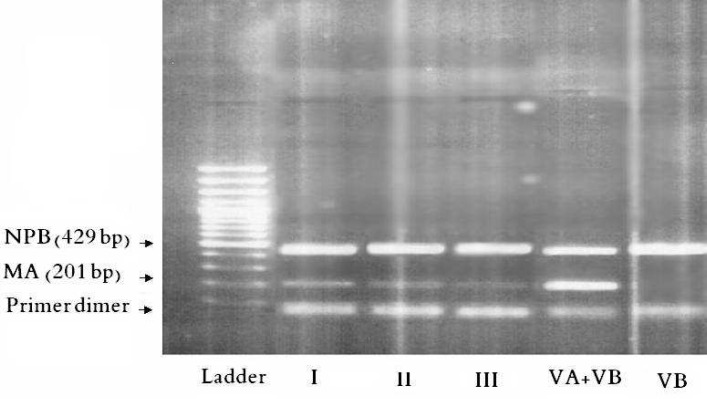
RT-PCR products gel electrphoresis of vrial RNA content extracted from same number of cells and FluV inoculants, exposed to POM-4960 by three different procedures (I, II, III). PCR reaction carried out using M primer (for detection of matrix protein gene) of FluVA and NP primer (for detection of nucleoprotein gene) of FluVB as internal control. I: PCR product of experiment I, II: PCR product of experiment II, III: PCR product of experiment III, VA+VB: PCR product of FluV-A&B, VB: PCR product of FluV-B.

**Fig 3:**
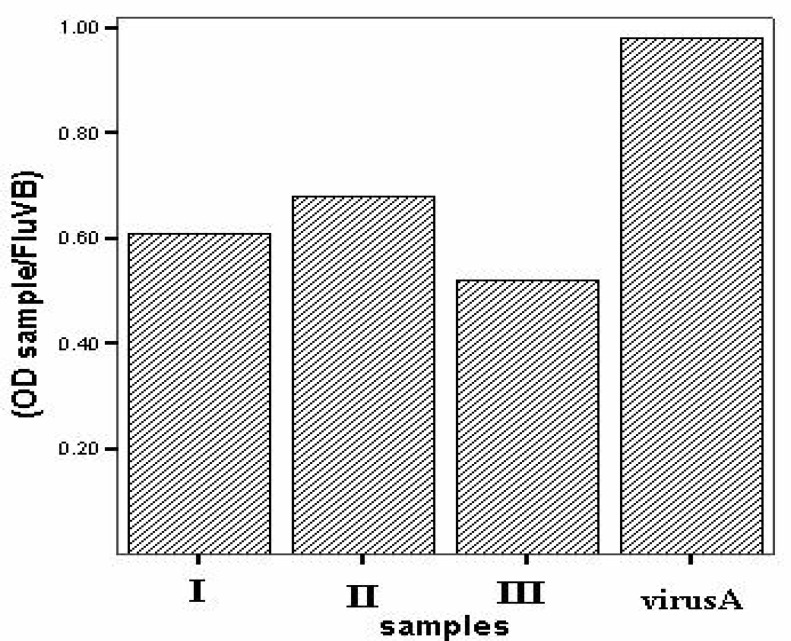
The viral genome content in treated samples was assessed by band densitometry. The results show meaningful difference (p<0.05) between OD ratio of genome content of treated samples with POM-4960 (with three different procedures: I, II, & III) compared to virus sample. FluV-B genome was used as internal control to normalize different test procedures.

Reduction in PCR product content could be obviously observed by a decrease in PCR product band density after running on gel (Fig.2). Semi-quantitative analysis on PCR products using band densitometry software “Image-Tool®” and statistical analysis by Mann-Whitney U test showed statically meaningful decrease (p<0.05) in genome content in the presence of POM-4960 (Fig.3).

**Direct immunofluorescent assay**

The expression of the FluV surface glycoprotein knobs on the infected cells (influenza CPE) was evident after one hour infection (control group), however in the presence of POM-4960 the viral fusion was suppressed and the fluorescence spots were markedly faded compared to the negative control. Hence, POM-4960 has shown a fusion inhibitory effect against influenza A virus. 

## Discussion

The previous studies on the chemical and biological properties of POMs have shown wide medical properties of POMs including anticancer and some enzyme inhibitory effects (19,20,21). In recent studies, the antiviral activity of POMs against several kinds of viruses in different cell lines has been mentioned (22). Among the POM compounds, POM-523 and POM-504 have been examined for their antiviral activity against FluV. These POMs have shown inhibitory effects against FluV replication via different mechanisms. In instance, negatively charged POM-504 could electrostatically prevent the attachment of HA1 molecules to the cell membrane receptors, whereas antiviral activity of POM-523 is based on fusion inhibition of HA2 into the cell membrane. 

In the current study, inhibitory effects of POM-4960 with Wells-Dawson structure against influenza virus A/H_1_N_1 _were examined. The low toxicity of POM-4960 in MDCK cell line could be confirmed by high differences (nearly 5-10 folds) among CC_50_ (100 µM/ml), MTC (50 µM/ml), and MEC_99_ (10µM/ml) concentrations. Hemagglutina-tion test showed high *in vitro *efficacy of POM-4960 against FluV which might reflect POM-4960 direct effects on HA molecules. In other words, POM-4960 considerably reduces binding of HA trimer to the cell receptors which are responsible for first stage of viral attachment. It is very likely that the negative charge of POM-4960 has an important role on its binding inhibition properties. This is quite similar to the negatively charged POM-504 which electrostatically interact with HA trimers. Our findings show that POM-4960 not only has binding inhibition ability in a way similar to POM-504, but also it inhibits viral fusion more like POM-523. Based on the experiments of “exposure to POM-4960 at 3 procedures (before-after and during) infection” (Table3), it could be concluded that POM-4960 protects the cells from binding and also inhibits fusion of viral particles into the cells. However, treating the cells with POM-4960 (before virus inoculation) is more effective than post-infection treatment. In order to analyze the POM-4960 target molecule in adsorption/penetration procedures, we also examined the inhibitory effect of this compound against binding of antibodies to viral membrane glycoprotein knobs. The results indicate that to some extent, POM-4960 can inhibit the interaction between glycoprotein knobs and antibodies. These findings were in good agreement with the results of complementary tests such as semiquantitative RT-PCR and direct immunofluorescent assay. A significant decrease in the fluorescence was observed following addition of POM-4960 (data not shown). 

In conclusion, POM-4960 is able to inhibit FluV-A virus replication in vitro. Overall, the results indicate that this new kind of POM, with dual mechanism of action (on binding and fusion of the virus) can be a good alternative to the previously invented POMs effective against FluV. Conducting in vivo studies on POM-4960 toxicology seem to be rational toward designing clinical trials for FluV infection treatment.
